# Metabolic Syndrome in Canadian Adults and Adolescents: Prevalence and Associated Dietary Intake

**DOI:** 10.5402/2012/816846

**Published:** 2012-11-20

**Authors:** Solmaz Setayeshgar, Susan J. Whiting, Hassanali Vatanparast

**Affiliations:** Division of Nutrition and Dietetics, College of Pharmacy and Nutrition, University of Saskatchewan, 110 Science Place, Thorvaldsen Building, Saskatoon, SK, Canada S7N 5C9

## Abstract

*Background.* Metabolic syndrome (MetS) includes five chronic disease risk factors which doubles the risk of CVD and increases the risk of diabetes fivefold. * Objective.* To determine the prevalence of MetS and its risk factors in Canadians (12–79 y) and to compare the dietary intake in Canadians with MetS and without MetS. * Subjects and Methods.* Cycle 1 of Canadian health measures survey, CHMS data, 2007–2009, was used. To identify MetS cases, the most recent criteria were used for adults and adolescents. Ethnical cut points for waist measurement were applied for adults. * Results and Conclusion.* The prevalence of MetS among 12–79 y Canadians was 18.31% with the lowest prevalence in adolescents (3.50%). Using ethnical cut points to define abdominal obesity increased the prevalence of MetS by 0.5% in adults. The most prevalent defining component of MetS in Canadians identified with MetS was abdominal obesity. Reduced HDL-C was equally prevalent among adolescents. Canadians with MetS consumed significantly more diet soft drinks, but less dairy products, dietary fat, and sugar-sweetened beverages compared to Canadians without MetS. Known cases of diabetes with MetS had healthier beverage choices compared to individuals without the diagnosis of diabetes, indicating adherence to nutrition recommendations.

## 1. Introduction

Mortality and morbidity due to cardiovascular disease (CVD) and diabetes are major public health concerns in Canada and worldwide. Metabolic syndrome (MetS) is a clustering of five chronic disease risk factors, including abdominal obesity, dyslipidemia (elevated triglycerides (TG) and reduced high-density lipoprotein cholesterol (HDL-C) level), hypertension, and elevated fasting blood glucose (FPG) [1]. MetS is considered to be the main contributor to CVD and diabetes [2, 3]. With MetS, the risk of CVD doubles and the risk of diabetes increases fivefold [1, 4]. Over the past 17 years (1992–2009), the prevalence of MetS in Canadian adults increased from 14.4% [2] to 19.1% [5]. 

Although dietary intake has been linked to individual components of MetS [6–9], dietary behaviour among Canadians with and without MetS has not been explored. The prevalence of MetS among Canadian adults, using different criteria, has been reported recently [5, 10]. However, the prevalence of MetS in adolescents as well as defining ethno-specific abdominal obesity in adults, using the most recent criteria, has yet to be explored. The most prevalent form of the constellation of metabolic abnormalities is found in patients with abdominal obesity [11].

The aims of our study are to determine the prevalence of MetS and its risk factors in Canadians (12–79 y) using nationally representative data, Canadian health measures survey (CHMS), and to compare the dietary intake in Canadians with MetS, and without MetS. As healthy dietary behaviour is an important factor in self-management of chronic diseases, it is likely that people with diagnosed disease such as diabetes adhere to a special diet [12]. Therefore, we compared the dietary intake between Canadians with MetS having diagnosed diabetes, to those with MetS having no diagnosis of diabetes.

## 2. Subjects and Methods

### 2.1. Study Population

Data from the Canadian Health Measures Survey (CHMS), Cycle 1, 2007–2009, conducted by Statistics Canada in partnership with Health Canada and Public Health Agency of Canada, was used. Statistics Canada, as a governmental organization, is “a member of the industry portfolio produces statistics that help Canadians better understand their country, its population, resources, economy, society, and culture. In addition to conducting a Census every five years, it conducts about 350 active surveys on virtually all aspects of Canadian life” [13]. Among those surveys CHMS is a nationally representative survey collecting health indicators among a sample of approximately 5,500 Canadians aged from 6 to 79 y (representative of 96.3% Canadians through multistage sampling strategy). The survey consists of two stages: the first stage is self-reported data collection through interviews, and the second section consists of taking direct physical measurements at Mobile Examination Centers (MEC). Individuals living on reserves or in other aboriginal settlements in the provinces, remote areas, institutional residents, and full-time members of the Canadian Forces were excluded from the survey. The sampling weights, provided by statistics Canada, were calculated by multiplying the selection weights for collection sites and the selection weights for dwellings (obtained from 2006 census), adjusted for nonresponse [14]. The final individual population weight was obtained after converting the household weights followed by adjustment for nonresponse at the interview stage and the MEC stage. For the purpose of our study, individuals under the age of 12, nonfasting participants, and pregnant women were excluded. The final unweighted number of respondents was 2,173 subjects.

### 2.2. Metabolic Syndrome

For adults (20–79 y), we applied the most recent unified definition which was established in 2005 by the International Diabetes Federation (IDF) in collaboration with American Heart Association/National Heart, Lung, and Blood Institute (AHA/NHLBI). The presence of at least three of the following five metabolic risk factors constitutes a diagnosis of MetS: abdominal obesity (Table 1 shows the cut points used in the current study) [15], elevated TG level (1.7 mmol/L), reduced HDL-C level (1.0 mmol/L in males; 1.3 mmol/L in females), elevated blood pressure (BP) (systolic ≥ 130 and/or diastolic ≥ 85 mm Hg), and elevated FPG level (≥5.6 mmol/L). Individuals who have already been diagnosed as hypertensive, diabetic, or those who were using antihypertensive drugs were also included. Since no Canadian study has considered ethno-specific cut points to define abdominal obesity which is highly recommended by International Diabetes Federation (IDF) recently [15], we apply that in our study.

In adolescents (12–19 y), age- and sex-specific cut points for each component (except for FPG), which were developed using the Third National Health and Nutrition Examination Survey (NHANES, from 1999 to 2002), were used to identify MetS [16]. Age- and sex-specific growth curves were created with the Lambda Mu Sigma method, and each MetS component growth curve was linked to the corresponding Adult Treatment Panel (ATP III) and IDF cut point. Each cut point reflects the midpoint of a given year (i.e., for age 12 the cut points represent 12.5 y) from 12 to 19 y and could be used for all adolescents within 1-year age range. In this study, the overall prevalence of MetS was reported using Adult Treatment Panel (ATP III) cut points for abdominal obesity in adolescents due to no significant difference in the prevalence of MetS using either ATP or IDF criteria.

### 2.3. Prevalence of MetS by Different Sociodemographic Characteristics

The age- and sex-specific groups in our analysis were males and females aged 12–19 y, 20–39 y, 40–59 y, and 60–79 y. Four levels of education, as the highest level achieved by any member of the household, were obtained, Table 2. Four economic status levels were based on the total household income and the number of individuals in the household. Only two groups of ethnicities, that is, White and non-White, were created due to few numbers of non-White individuals in various subgroups. Daily smokers, occasional smokers, and those who stopped smoking for less than a year, were considered as smokers. Physical activity was measured in CHMS using a questionnaire which calculated the total daily leisure time energy expenditure (EE) values (kcal/kg/day). Respondents were subsequently categorized into “active” (EE ≥ 3), “moderate” (1.5 ≤ EE < 3), or “inactive” (0 ≤ E < 1.5) physical activity.

### 2.4. Dietary Assessment

In CHMS, usual dietary intake was collected through a semiquantitative food-frequency questionnaire. Dietary intake was collected based on the frequency of daily, weekly, monthly, or yearly consumption. Food groups in CHMS were defined as follows: meat and fish (e.g., red meat, organs, hotdogs, sausage or bacon, sea foods, eggs, beans, and nuts), grains, fruit, and vegetable (e.g., hot/cold cereal, white bread, brown bread, any kind of rice, any kind of pasta, fruit, and vegetable including potato), milk and dairy product (e.g., milk, cottage cheese, and yogurt or ice cream), dietary fat (regular-fat salad dressing or mayonnaise, and regular-fat potato chips, tortilla chips, or corn chips), water and soft drink (e.g., regular soft drink, sport drink, and fruit drink, diet soft drink, fruit/vegetable juice). The questions for each section were only included the frequency of consumption. The respondents were able to mention the frequency of consumption per day, week, month, or year. “Do not know” and “Refused” were not allowed [17]. Except the section on water and soft drink consumption all other dietary intake questions were made for the first time for CHMS. Water and soft drink consumption questions were derived from the National Population Health Survey, NPHS, cycle 6 [18]. Validation of questionnaire responses was made at the end of completing data collection at each site. A respondent's case file was reviewed and adjusted using notes and remarks made by interviewers. The food groups used in this study are consistent with what was used in CHMS. For the purpose of the current study all dietary consumption data were converted into a daily frequency of consumption (times/day).

### 2.5. Diabetes

We hypothesized that having been already diagnosed with chronic diseases such as diabetes might have impact on dietary intake, and such individuals might adhere to a special diet. Therefore, we compared the dietary intake in Canadians identified with MetS and having diagnosed diabetes with Canadians identified with MetS having no diagnosis of diabetes. Data on self-reported diabetes, which was specified as being diagnosed by health professionals, is available in CHMS. We used this indicator to identify the difference in dietary intake between individuals with MetS and diagnosed diabetes, and individuals with MetS who are not diagnosed with diabetes. The latter group consisted of different two subgroups: (i) individuals with MetS who had glycated hemoglobin (HbA1c) levels ≥6.5% (criterion for diabetes) [19] yet without the knowledge of their disease state and (ii) individuals with MetS and without diabetes, that is, glycated hemoglobin (HbA1c) levels <6.5%. In 2008, an international committee with members of American Diabetes Association, the European Association for the Study of Diabetes, and the International Diabetes Federation suggested using HbA1c [19] as an accurate and precise measure for chronic glycemic levels and well-correlated criteria with the risk of diabetes complications in both adults and adolescents [19].

### 2.6. Data Analysis

Simple binary logistic regression was used to estimate the crude significant difference in the distribution of MetS by each sociodemographic variable. We also looked at the overlap of 95% confidence interval to interpret the significant difference between the levels of each sociodemographic variable. The frequency of dietary consumption was evaluated in individuals with and without MetS using mean intake values. To determine significant differences in dietary intake between individuals with and without MetS or with diagnosed diabetes and no diagnosis, the independent sample *t*-test was used.

Data manipulation, cleaning, and creation of new variables were done using PASW statistics 19. All statistical analyses were conducted by STATA SE 11. As per Statistics Canada's recommendation, all analyses were weighted and bootstrapped to obtain estimates that are representative of the Canadian population. The degrees of freedom of 11 in CHMS Cycle 1, due to sampling structure, were considered in analyses. Alpha was set at 0.05.

## 3. Results

The CHMS participants in our study (*n* = 2173) represent 27 043 753 Canadians aged 12–79 y. The estimated overall prevalence of MetS for this group was 18.31% with no significant difference between males and females (17% versus 19%, resp.). According to 95% confidence interval, the prevalence of MetS (Table 2) was greater in individuals aged 40–59 y and 60–79 y (22%, 41%, resp.) compared to either 12–19 y (3.5%) or 20–39 y (7.8%).

**Figure 1:**
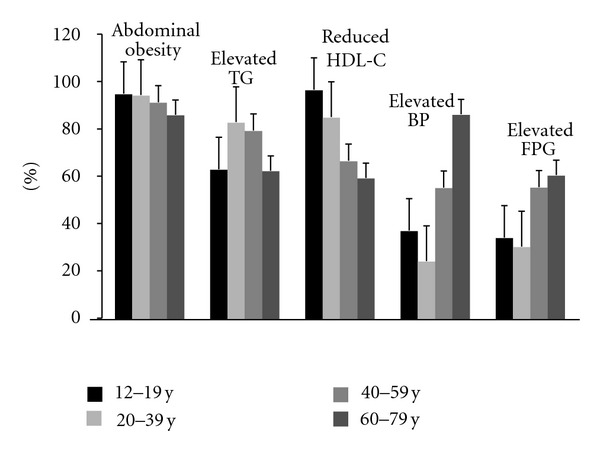
Age-specific prevalence of the metabolic syndrome components among individuals identified with metabolic syndrome aged 12 to 79 y (*n* = 2173). Canadian Health Measures Survey, Cycle 1, 2007–2009. TG: triglyceride, BP: blood pressure, FPG: fasting plasma glucose.

Figure 1 illustrates the age-specific distribution of MetS components among individuals identified with MetS. Abdominal obesity was the most frequent component of MetS in almost all age groups (~86% to ~95%) although the prevalence decreased by increase in age due to high prevalence of other components in older individuals. In adolescents, reduced HDL-C and abdominal obesity were almost equally the most prevalent components (94.9% and 96.5%, resp.). Among 60–79 y individuals, elevated BP and abdominal obesity had similar prevalence (~86%). Elevated TG and FPG were more frequent in 20–39 y (83%) and 60–79 y (60.5%) individuals, respectively.

By looking at the 95% confidence interval, the prevalence of MetS was significantly lower among Canadian households with some postsecondary education or postsecondary graduation compared to both less than secondary education and secondary school graduation (*P* < 0.05, Table 2). Moreover, the prevalence of MetS was lower among active Canadian compared to moderately active and inactive counterparts (*P* < 0.05, Table 2).

Canadians with MetS significantly consumed less dairy products, dietary fat, sugar-sweetened beverages (SSBs), and more diet soft drinks compared to Canadians without MetS (*P* < 0.05, Table 3). Canadians with MetS and diagnosed diabetes significantly consumed less fruit and vegetable juice (*P* = 0.04, Table 3) compared to individuals with no diagnosis of diabetes.

## 4. Discussion

The prevalence of MetS was 18% among Canadians aged 12–79 y with a higher prevalence rate in older Canadians and with no significant difference between sexes. The prevalence of MetS was lower among physically active Canadians than inactive or moderately active individuals or among Canadians with higher levels of education compared to individuals with lower level of education. Abdominal obesity was identified using different ethno-specific cut points for Canadian adults. Considering Canadians identified with MetS abdominal obesity in all age groups was the most prevalent component. In adolescents, reduced HDL-C was equally the most prevalent component of MetS as abdominal obesity.

Lower consumption of SSBs and greater consumption of diet soft drinks in Canadians identified with MetS led to investigate the effect of the possibility of having been diagnosed with diabetes in individuals with MetS on adherence to a special diet. Although the SSBs and diet soft drink consumption was lower and greater in diabetics, respectively, the only statistically significant difference was observed in lower consumption of fruit and vegetable juice. Moreover, Canadians with MetS consumed less dairy products and dietary fat.

A similar trend in the prevalence of MetS regarding age in our study was observed among US population using NHANES data [20, 21] wherein prevalence increased from 6.7% in individuals aged 20–29 y to ~43% in 60 and over. The greater prevalence of MetS in older adults is not surprising as this group showed higher prevalence of most MetS defining components (Figure 1).

The majority of MetS studies have focused on adults; however, identifying MetS risks in adolescents is important in preventing the early establishment of diseases related to MetS. The reason for not including adolescents in such studies is due to the lack of consistent defined criteria of MetS [15]. Using the age- and sex-specific criteria, the prevalence of MetS in American adolescents was 7.6% [16] compared to 3.5% in our study. The prevalence in American adolescents is expected to double because the obesity prevalence is twice among American adolescents (18.1%) [22] compared to Canadian adolescents (9.4%) [23].

Including adolescents to the data in our study decreased the previously reported [5] overall prevalence of 19.6% (the prevalence in adults over 18 y) to 18.31% due to the lower prevalence of MetS in adolescents. Using the same criteria and the same data, the estimated prevalence of MetS in Canadian adults over 18 y in our study was similar to Riediger and Clara's [5] report (19.6% versus 19.1%, resp.) with a 0.5% increase in our study. Using ethno-specific cut points in our study increased the prevalence of abdominal obesity in each age group by 1 to 4% among adults (Table 2). Therefore, the 0.5% difference in the prevalence for adults at population level is due to using ethno-specific defined abdominal obesity.

Abdominal obesity was found the most frequent component of MetS in almost all age groups identified with MetS in our study. Additionally, abdominal obesity was the most prevalent metabolic abnormality not only in individuals identified with MetS but also in the whole population (35.1%, data not shown) which is consistent with US population data [20, 24]. There is enough evidence supporting the notion that abdominal obesity is predictive of metabolic risk factors [11]. It has been previously observed that abdominal obesity is associated with reduced HDL-C and Low Density Lipoprotein Cholesterol (LDL-C) size, increased LDL-C number [25, 26], triglyceride [25], and insulin resistance [27, 28]. In adolescents, reduced HDL-C was the most prevalent component (96%) similarly to American adolescents [29]. One reason could be due to biological changes in which mean HDL-C levels drop especially among boys over 12 y during puberty in most ethnic groups [30, 31]. Lower prevalence of MetS among adolescents due to lower prevalence of risk factors along with abdominal obesity and reduced HDL-C as the most prevalent components could help to characterise a strategy to decrease the prevalence of MetS in adulthood.

Studies have examined the association between dairy intake and risk of developing MetS, type 2 diabetes, and cardiovascular disease [6, 32, 33]. Lutsey et al. reported individuals with highest quintile of dairy consumption were at 13% lower risk of developing MetS [6]. Using NHANES 1999–2004 data (8970 women, 8091 men aged ≥18 y), a significant inverse association between consumption of dairy products particularly, milk and yogurt, was seen with obesity, abdominal obesity, and MetS [33]. We found that Canadians with MetS had less diary product consumption than individuals without MetS. One of the defining components of MetS in some MetS cases (*n* = 71) was the existence of diabetes. Among MetS cases, although individuals with diabetes consumed greater dairy products, the consumption was not statistically significant from the individuals without diabetes probably due to the small number of MetS cases with diabetes.

The most specific dietary recommendations for individuals with diabetes are made for focusing on carbohydrate, dietary fat, and cholesterol intake [34]. Evidence suggests that dietary fat quality influences insulin sensitivity and its associated metabolic abnormalities [35]. Individuals with MetS consumed less dietary fat compared to individuals without MetS. However, among MetS cases, diabetic individuals did not consume significantly less dietary fat than individuals with nondiabetics. Our finding is comparable to the results from the Multiethnic Cohort Study (MEC) [34] conducted in five ethnic groups (*n* = 13 776). Saturated fat consumption was not significantly different between diabetics and individuals without diabetes. Individuals with MetS, as a syndrome defined by specific risks, might not adhere to special diet, hypothetically due to unawareness about their situation. In contrast, individuals with diagnosed chronic conditions likely adhere to a special diet. Although we did not find difference in fat intake between MetS cases with and without diagnosed diabetes, whether such difference exists with other chronic conditions, such as cardiovascular disease, needs further investigation.

Lower consumption of juice (>10% difference) in the MEC study was observed in participants with diabetes compared to those without [34]. This observation may reflect the adherence of individuals with a diagnosis of diabetes to one of the nutritional recommendations provided by Canadian Diabetes Association “have vegetables and fruit more often than juice” [36]. Indeed, diabetics are reported to pay more attention to the food labels especially to the sugar information, than those without diabetes [12].

There is evidence that diet soft drinks are usually consumed more by individuals who are following other healthy behaviors [37]. The MEC study [34] observed that diabetic individuals on average consumed significantly 2.6 times more diet soft drinks than individuals without diabetes, whereas the consumption of regular soft drinks was less than half of what individuals without diabetes drank [34]. This observation was not statistically significant in our study, possibly due to the small subsamples of participants included in the analyses. Moreover, the MEC study and our study are different by virtue of ethnicity of participants and sample size.

### 4.1. Limitations

The fasted subsample used in our study was smaller than the whole CHMS sample. To generalize data to the Canadian population, we used the specific weights provided in CHMS for fasted subsample. Although CHMS collected usual dietary intake through semiquantitative questionnaire, the quantity of the consumption is not provided to obtain an exact measure of dietary intake.

## 5. Conclusion

The prevalence of MetS among Canadians age from 12 to 79 y was 18.3%, with the lowest prevalence in the youngest group (3.5%) and the highest among those 60–79 y (41%). The prevention strategy for Canadian population needs to consider those individuals who are less educated or who are less active or both. Additionally, to define abdominal obesity it would be more precise to implement ethno-specific cut points while calculating the prevalence of MetS. Canadians with MetS reported drinking significantly more diet soft drinks and less dairy products, dietary fat, and regular soft drinks compared to Canadians without MetS. Comparing the dietary intake of individuals with no diagnosis of diabetes, the dietary intake of individuals with diagnosed diabetes and MetS; particularly low consumption of sweetened beverages and juice may reflect adherence to health messages in diabetic patients. The latter observation needs more investigation considering greater sample size and other chronic diseases including cardiovascular diseases.

## Figures and Tables

**Table 1:** Waist circumference threshold for abdominal obesity.

Population	Men	Women
White (Canadian, American, and European)	≥102 cm	≥88 cm
Middle East, Mediterranean, Sub-Saharan African, and West Asian	≥94 cm	≥80 cm
Asian and Latin American	≥90 cm	≥80 cm
Chinese	≥85 cm	≥80 cm
Japanese	≥85 cm	≥90 cm

**Table 2:** Weighted estimates of the prevalence of identified metabolic syndrome by sociodemographic characteristics of Canadians aged 12 to 79, Canadian Health Measures Survey, Cycle 1, 2007–2009 (*n* = 2173).

Characteristics	MetS, estimated prevalence (SE^1^)	Confidence intervals (CIs)
Sex		
Male	17.25 (1.8)	13.26–21.25
Female	19.34 (1.7)	15.59–23.08
Age group		
12–19 y^2^	3.5 (1.5)^F^	0.20–6.89
20–39 y	7.8 (1.4)^E^	4.74–10.97
40–59 y	21.6 (2.5)*	16.17–27.12
60–79 y	40.9 (3.5)*	33.05–48.77
Education level		
Less than secondary school graduation^2^	39.7 (6.9)^E^	24.49–55.06
Secondary school graduation	31.1 (4.1)	22.03–40.26
Some postsecondary	10.7 (3.3)^∗E^	3. 36–18.16
Postsecondary graduation	14.6 (1.4)*	11.53–17.66
Income level		
Lowest income^2^	19.9 (4.6)^E^	9.69–30.13
Lower-middle income	29.7 (4.3)	20.16–39.33
Upper-middle income	20.3 (2.2)	15.51–25.21
Highest income	14.1 (1.5)	10.69–17.47
Physical activity		
Inactive^2^	23.0 (2.0)	18.45–27.55
Moderately active	17.1 (1.6)*	13.55–20.72
Active	8.7(1.5)^∗E^	5.27–12.29
Alcohol		
Never drink	17.6 (4.5)^E^	7.57–27.68
Ever drink	18.3 (1.5)	15.10–21.65
Ethnicity		
Non-White	21.2 (3.8)^E^	12.70–29.70
White	17.7 (1.3)	14.84–20.59
Smoking		
Nonsmokers	18.4 (1.6)	14.87–22.04
Smokers	17.7 (1.6)	14.12–21.41

^1^SE: standard error.

^2^Reference level.

^F^Data with a coefficient of variation >33.3%. The user is advised that 3.5% do not meet Statistics Canada's quality standards for this statistical program.

^E^Data with a coefficient of variation from 16.6% to 33.3%.

*Significant (*P* < 0.05), simple binary logistic regression.

**Table 3:** Dietary consumption among Canadians aged from 12 to 79 y with and without metabolic syndrome (MetS), Canadian Health Measures Survey, Cycle 1, 2007–2009 (*n* = 2173).

	Individuals without MetS	Individuals with MetS	Individuals with MetS and diagnosed diabetes	Individuals with MetS and no diagnosis of diabetes
Food and beverages times/day^1^	Mean (SE^2^) CIs^3^	Mean (SE^2^) CIs^3^	Mean (SE^2^) CIs^3^	Mean (SE^2^) CIs^3^
	*n* = 1746	*n* = 426	*n* = 71	*n* = 355
Meat and fish				
Red meat, organs, hotdogs, sausage or bacon, seas foods, eggs, beans, and nuts	1.92 (0.17)	1.55 (0.16)	1.27 (0.06)	1.60 (0.20)
	1.54–2.31	1.19–1.91	1.13–1.42	1.15–2.05

Grains, fruit and vegetable				
(i) Hot/cold cereal, white bread, brown bread, rice, pasta (grains)	3.18 (0.21)	3.6 (0.31)	4.62 (0.90)	3.41 (1.34)
	2.72–3.64	2.91–4.30	2.63–6.62	2.65–4.17
(ii) Fruit and vegetable	3.71 (0.08)	3.61 (0.17)	3.64 (0.25)	3.60 (0.18)
	3.52–3.90	3.21–3.99	3.08–4.20	3.18–4.01

Milk and dairy products				
Milk, cottage cheese, and yogurt or ice cream	1.69 (0.05)*	1.38 (0.07)*	1.55 (0.10)	1.34 (0.08)
	1.56–1.82	1.21–1.54	1.33–1.77	1.16–1.52

Dietary fat				
Regular-fat salad dressing or mayonnaise and regular-fat potato chips, tortilla chips, or corn chips	0.48 (0.02)*	0.36 (0.03)*	0.39 (0.14)	0.36 (0.02)
	0.44–0.53	0.29–0.43	0.07–0.71	0.31–0.40

Water and soft drinks				
(i) Regular soft drink, sport drink, and fruit drink (sugar-sweetened beverages )	0.53 (0.04)*	0.04)*	0.20 (0.07)	0.40 (0.05)
	0.44–0.62	0.27–0.46	0.04–0.35	0.28–0.52
(ii) Diet soft drink	0.14 (0.02)*	0.22 (0.03)*	0.47 (0.13)	0.17 (0.03)
	0.10–0.18	0.16–0.28	0.18–0.76	0.09–0.26
(iii) Fruit and vegetable juice	0.74 (0.04)	0.70 (0.06)	0.50 (0.07)^†^	0.74 (0.07)^†^
	0.65–0.82	0.55–0.85	0.34–0.65	0.57–0.91

^1^Frequency of consumption.

^2^SE: standard error.

^3^Confidence intervals.

^∗, †^Significant (*P* < 0.05), Independent samples *t*-test.
